# Identifying Diagnostic Markers and Constructing Predictive Models for Oxidative Stress in Multiple Sclerosis

**DOI:** 10.3390/ijms25147551

**Published:** 2024-07-10

**Authors:** Yantuanjin Ma, Fang Wang, Qiting Zhao, Lili Zhang, Shunmei Chen, Shufen Wang

**Affiliations:** 1 Institute of Biomedical Engineering, Kunming Medical Univesity, Kunming 650500, China; 20190329@kmmu.edu.cn (Y.M.); zqt940601@126.com (Q.Z.); z13889646862@163.com (L.Z.); 2 Department of Science and Technology, Kunming Medical University, Kunming 650500, China; fangwang00@sina.com

**Keywords:** multiple sclerosis, oxidative stress, diagnostic biomarkers, immune cell infiltration, molecular docking

## Abstract

Multiple sclerosis (MS) is a chronic disease characterized by inflammation and neurodegeneration of the central nervous system. Despite the significant role of oxidative stress in the pathogenesis of MS, its precise molecular mechanisms remain unclear. This study utilized microarray datasets from the GEO database to analyze differentially expressed oxidative-stress-related genes (DE-OSRGs), identifying 101 DE-OSRGs. Gene Ontology (GO) and Kyoto Encyclopedia of Genes and Genomes (KEGG) analyses indicate that these genes are primarily involved in oxidative stress and immune responses. Through protein–protein interaction (PPI) network, LASSO regression, and logistic regression analyses, four genes (*MMP9*, *NFKBIA*, *NFKB1*, and *SRC*) were identified as being closely related to MS. A diagnostic prediction model based on logistic regression demonstrated good predictive power, as shown by the nomogram curve index and DAC results. An immune-cell infiltration analysis using CIBERSORT revealed significant correlations between these genes and immune cell subpopulations. Abnormal oxidative stress and upregulated expression of key genes were observed in the blood and brain tissues of EAE mice. A molecular docking analysis suggested strong binding potentials between the proteins of these genes and several drug molecules, including isoquercitrin, decitabine, benztropine, and curcumin. In conclusion, this study identifies and validates potential diagnostic biomarkers for MS, establishes an effective prediction model, and provides new insights for the early diagnosis and personalized treatment of MS.

## 1. Introduction

Multiple sclerosis (MS) is a chronic inflammatory condition that predominantly impacts the central nervous system, and it is a major cause of neurological impairment and disability [1]. Clinically and pathologically, MS is characterized by episodes of inflammation, demyelination, and axonal damage, which, ultimately, result in impaired neural transmission and loss of neurological functions [2]. Pathologically, MS is distinguished by the loss of myelin sheaths, injury to nerve fibers, and proliferation of glial cells [3]. These pathological changes primarily affect the white and gray matter regions within the central nervous system, particularly in the brain and spinal cord [3].

MS and its complications significantly impact both the physical and mental health of affected individuals, and they also pose a substantial economic burden to society [4]. The number of patients with MS has been increasing globally, especially in China, where the incidence of MS has been on the rise in recent years [5]. Currently, primary treatment options for MS in the United States and other countries include immunomodulatory therapy, corticosteroid therapy, and symptomatic management [6]. Although these therapies can decelerate disease progression, no cure or definitive treatment for MS currently exists [7]. Therefore, there is an urgent need for further research into the pathophysiological mechanisms of MS to identify reliable biomarkers for early diagnosis and therapeutic intervention.

Oxidative stress (OS) is considered to be intricately linked to the progression of MS [8]. Elevated oxidative stress levels can result in the production of reactive oxygen species (ROS) through mechanisms including mitochondrial dysfunction, inflammatory responses, and immune system activation [9]. This oxidative stress leads to myelin damage, axonal degeneration, and neuroinflammation, thus aggravating central nervous system damage [10,11]. Research indicates that ROS are common elements in numerous signaling pathways, ultimately causing damage to various target organ systems [12]. ROS encompass molecular oxygen and its derivatives, nitric oxide (NO), hypochlorous acid (HOCl), peroxynitrite (ONOO−), hydrogen peroxide (H_2_O_2_), hydroxyl radicals (HO ), superoxide anions (O_2−_), and lipid radicals [13]. Many ROS are free radicals, possessing unpaired electrons. An overabundance of ROS can overwhelm the body’s endogenous antioxidant defense systems, leading to the oxidation of biomolecules, such as DNA, proteins, carbohydrates, and lipids, and resulting in an oxidative stress state [14,15]. Nevertheless, research on the role of oxidative stress in the pathogenesis of MS is still relatively scarce.

In order to delve deeper into the pathogenesis of multiple sclerosis (MS), particularly the involvement of oxidative-stress-related genes, we conducted an analysis of differentially expressed oxidative-stress-related genes (DE-OSRGs) between patients with MS and healthy controls using microarray datasets from the GEO database. Through the utilization of protein–protein interaction (PPI) analysis and machine learning algorithms, we identified and screened potential diagnostic biomarkers for MS and, subsequently, developed a diagnostic prediction model using logistic regression techniques. Our research uncovered several candidate genes strongly associated with immune infiltration, which hold promise for integration into diagnostic prediction models for MS, thereby offering novel perspectives for its treatment. In essence, this study aims to provide fresh insights and potential therapeutic avenues for the early diagnosis and personalized management of MS.

## 2. Result

### 2.1. Identification of DE-OSRGs in Multiple Sclerosis

**Figure 1:**
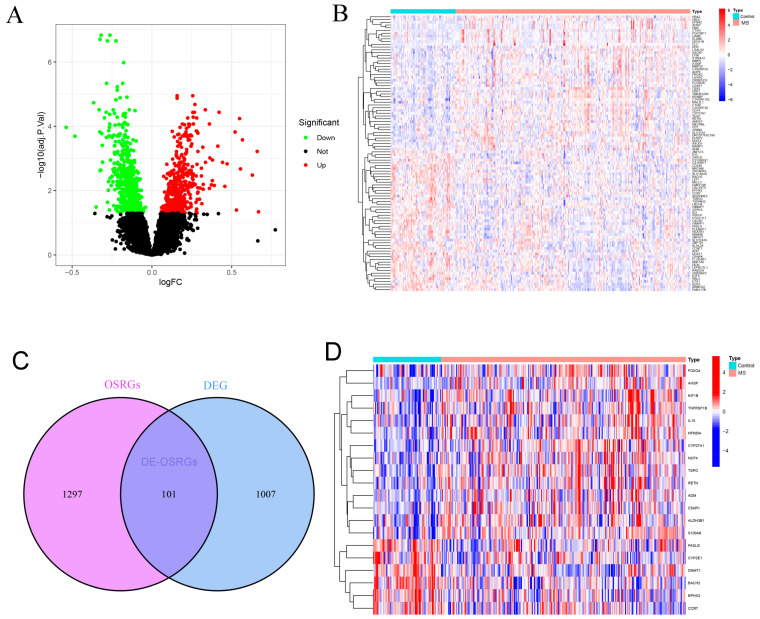
Differential expression analysis between MS and normal samples: (**A**,**B**) volcano plots and heatmaps illustrate genes exhibiting differential expression; (**C**) overlapping genes between DEGs and DE-OSRGs; (**D**) heatmap illustrating significantly differentially expressed OSRGs.

The GEO GSE136411 microarray expression profile comprises 243 MS samples and 68 control samples. An analysis indicated 1108 DEGs between the two groups, with 470 genes upregulated and 638 genes downregulated (Figure 1A). The heatmap illustrates the top 100 most significant DEGs (Figure 1B). Furthermore, 101 overlapping genes between OSRGs and DEGs were identified (Figure 1C), with a heatmap displaying the top 20 significantly different DE-OSRGs (Figure 1D). These results demonstrate significant differences in gene expression between patients with MS and controls, suggesting that oxidative stress may play an important role in MS.

### 2.2. Functional Enrichment Analysis of DE-OSRGs in Multiple Sclerosis

**Figure 2:**
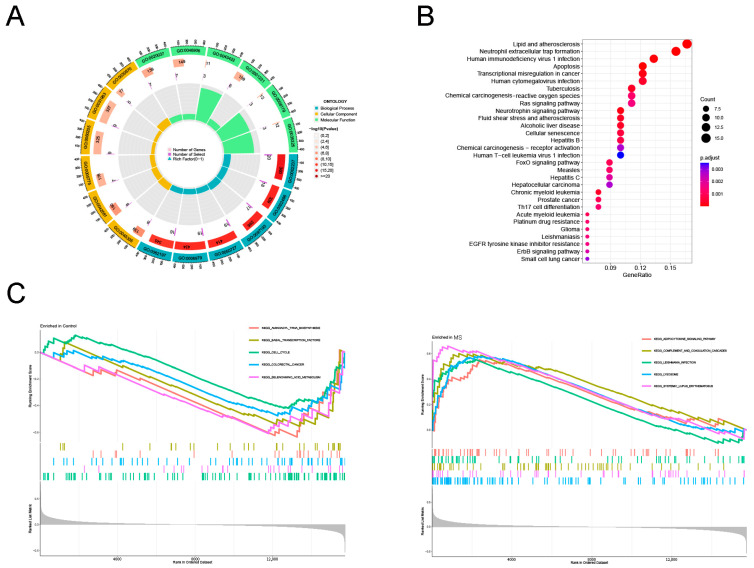
Functional enrichment analysis for DE-OSRGs: (**A**) GO terms enriched in Biological Process (BP), Cellular Component (CC), and Molecular Function (MF) of the DE-OSRGs; (**B**) KEGG pathway enrichment analysis of the DE-OSRGs; (**C**) GSEA-identified biological pathways associated with the development of MS.

To explore the potential functional relationships of these DE-OSRGs, we conducted a functional analysis using “clusterProfiler”, including GO and KEGG analyses. The results revealed a total enrichment of 879 GO terms, including 794 biological processes (BPs), 25 cellular components (CCs), and 60 molecular functions (MFs) (Figure 2A). These biological processes mainly involve the intrinsic apoptotic signaling pathway, regulation of inflammatory response, and response to oxidative stress. The molecular functions primarily include adenylate cyclase binding and Toll-like receptor binding. Regarding the cellular components, they mainly include phagocytic vesicles and the NADPH oxidase complex. The KEGG analysis showed that DE-OSRGs are mainly enriched in lipids, as well as atherosclerosis, neutrophil extracellular trap formation, chemical carcinogenesis—reactive oxygen species, apoptosis, and the Ras signaling pathway (Figure 2B). Additionally, to uncover the potential biological mechanisms of MS caused by sepsis, we performed a gene set enrichment analysis (GSEA) involving gene sets from MSigDB. The results suggest that immune response and metabolism may play significant roles in the development of MS (Figure 2C).

### 2.3. Identification of Diagnostic Genes for Multiple Sclerosis

**Figure 3:**
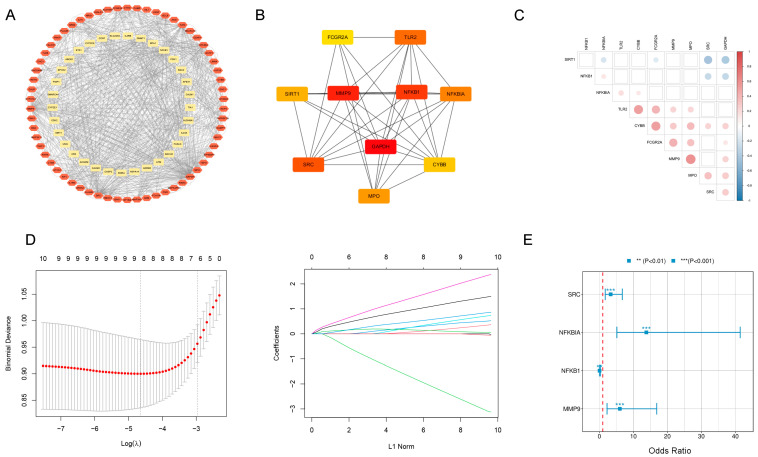
Identifying hub genes associated with oxidative stress in the pathogenesis of MS: (**A**) construction of the PPI network for the DE-OSGRs using the STRING database; (**B**) central genes identified through Cytohubba screening in the network, where darker colors (red) indicate higher scores; (**C**) Pearson correlation analysis of the 10 central genes; (**D**) LASSO regression, coupled with 10-fold cross-validation for penalty parameter tuning, identified eight feature genes; (**E**) a logistic regression model was used to identify feature genes with statistically significant differences in “riskScores”.

To evaluate the potential of the DE-OSRGs as diagnostic markers for DN, cytoHubba, machine learning, and logistic regression methods were used to identify candidate genes. First, to identify hub genes (those with the strongest interactions with other DE-OSRGs), a protein–protein interaction (PPI) network was constructed using the STRING database (Figure 3A) and analyzed with Cytoscape, resulting in a network containing 10 candidate genes (*FCGR2A*, *TLR2*, *SIRT1*, *MMP9*, *NFKB1*, *NFKBIA*, *GAFDH*, *SRC*, *CYBB*, and *MPO*) (Figure 3B). Positive correlations were found among *TLR2*, *CYBB*, *FCGR2A*, *MMP9*, and *MPO*, whereas *SRC* showed negative correlations with *SIRT1* and *GAPDH* (Figure 3C). Next, LASSO regression was used to select 8 MS-related features, and the penalty parameters were optimized through 10-fold cross-validation (Figure 3D). Finally, logistic regression analysis using the R package (version 4.4.1) “glm” was conducted on these eight marker genes, identifying four statistically significant risk genes as the final hub genes. In the GES3234 dataset, *MMP9, NFKBIA*, and *SRC*, excluding *NFKB1*, were found to be independent risk factors for MS development (Figure 3E).

### 2.4. Establishment and Evaluation of Multiple Sclerosis Diagnostic Prediction Model

**Figure 4:**
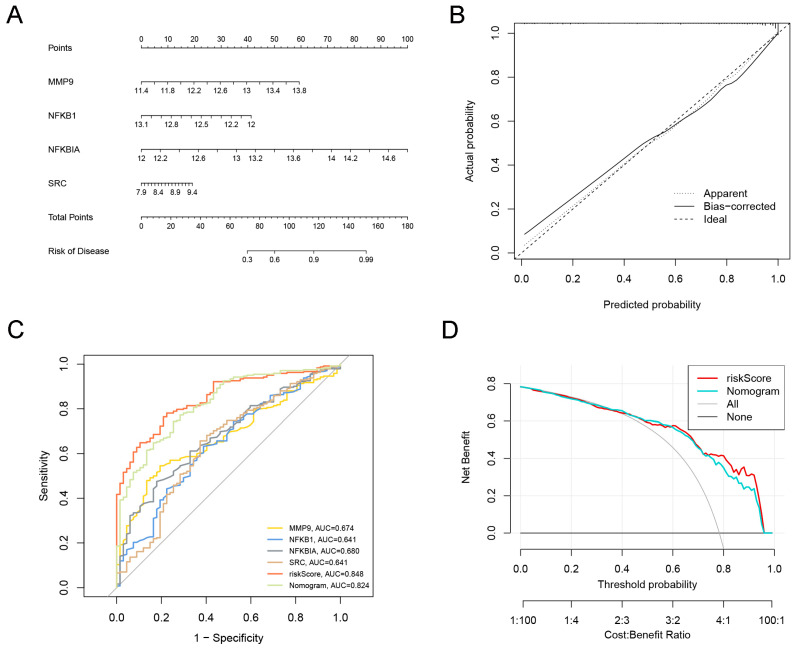
Establishment of predictive and diagnostic models for MS: (**A**) construction of nomogram model based on hub genes; (**B**) calibration curve of the nomogram prediction; (**C**) ROC curves for feature genes, riskScores, and the nomogram model; (**D**) DAC curves evaluating the accuracy of riskScores and the nomogram model.

**Figure 5:**
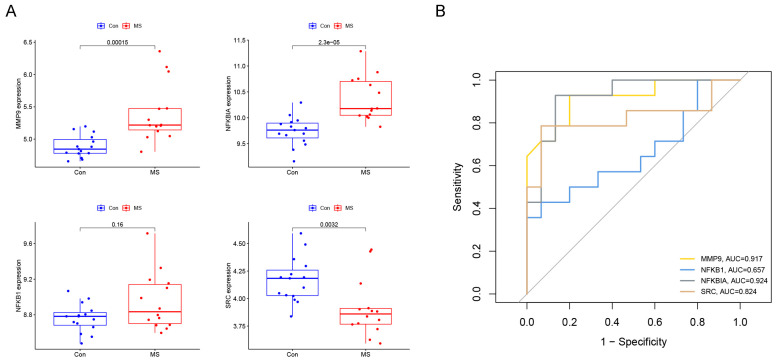
Validation of expression and predictive capability of feature genes in the GSE21942 dataset: (**A**) expression levels of the four characteristic genes; (**B**) validation of the ROC curves for the four feature genes.

To further understand the role of hub genes in MS diagnosis and prediction, a nomogram corresponding to MS diagnosis and prediction models was constructed by combining the genes *MMP9*, *NFKBIA*, *SRC*, and *NFKB1* with the risk scores (Figure 4A). The calibration curve of the incidence of MS shows that the actual incidence rate was highly consistent with the incidence rate predicted by the nomogram, indicating excellent predictive value (Figure 4B). The ROC results show that the nomogram model had an AUC of 0.824, demonstrating high diagnostic feasibility (Figure 4C). The DCA results show that the combined diagnostic model predicted MS incidence better than individual diagnostic genes (Figure 4D), further confirming the model’s superiority. Additionally, the predictive performances of the *MMP9*, *NFKBIA*, *SRC*, and *NFKB1* genes were validated using the GSE21942 dataset. Figure 5A illustrates the expression patterns of these genes in MS, revealing that *MMP9*, *NFKBIA*, and *NFKB1* were significantly upregulated in the MS samples, while *SRC* was increased in normal samples. A subsequent ROC curve analysis demonstrated that the new model had high accuracy in distinguishing normal samples from MS samples, with an AUC of 0.946 (Figure 5A). Furthermore, ROC curves were plotted for these four marker genes to reveal each gene’s diagnostic ability in distinguishing MS samples from normal samples. Except for *NFKB1*, the AUC values of the other three genes were all above 0.8 (Figure 5B). These results indicate that hub genes are practically feasible and reliable for diagnosing MS.

### 2.5. Expressions of the Four Characteristic Genes in the EAE Model

**Figure 6:**
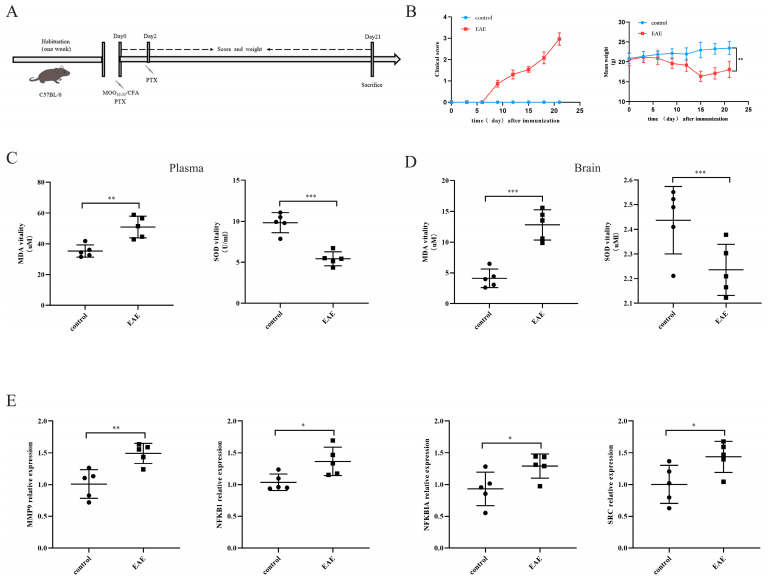
Oxidative stress indicators and expression levels of the 4 hub genes in the EAE model: (**A**) flow diagram illustrating the EAE mouse model; (**B**) clinical scores and weight changes in the EAE mice; (**C**,**D**) SOD and MDA activities in plasma and brain tissue; (**E**) expressions of the mRNA levels of the four feature genes in the EAE mice. Compared with the control group: * *p* < 0.05, ** *p* < 0.01, and *** *p* < 0.001.

The EAE model is an important tool for studying MS, effectively simulating the pathology and clinical features of MS. Compared to normal mice, EAE mice induced with MOG_35–55_ exhibit severe clinical symptoms and significant weight loss (Figure 6B). To further understand the impact of oxidative stress response in the EAE mouse model, the levels of MDA and SOD in serum and brain tissue were measured. The results show that the activity of MDA in the serum and brain tissue of EAE mice was significantly higher than that of normal control mice, while the activity of SOD was significantly inhibited (Figure 6C,D). This indicates a disruption of the oxidative stress in the MOG_35–55_-induced EAE mouse model. Furthermore, to verify the expressions of the *MMP9*, *NFKBIA*, *NFKB1*, and *SRC* genes in the EAE model, qPCR was used to detect the transcription levels of these four hub genes in brain tissue. As expected, the expressions of *MMP9*, *NFKBIA*, *NFKB1*, and *SRC* were significantly higher in the EAE mice compared to the normal control mice (Figure 6E). In summary, the EAE model successfully simulates the main pathological features of MS, particularly the imbalance in the oxidative stress response, and reveals significant upregulations of the *MMP9*, *NFKBIA*, *NFKB1*, and *SRC* genes, suggesting that these genes may play important roles in the pathology of MS.

### 2.6. Association between Characteristic Genes and the Proportion of Infiltrating Immune Cells

**Figure 7:**
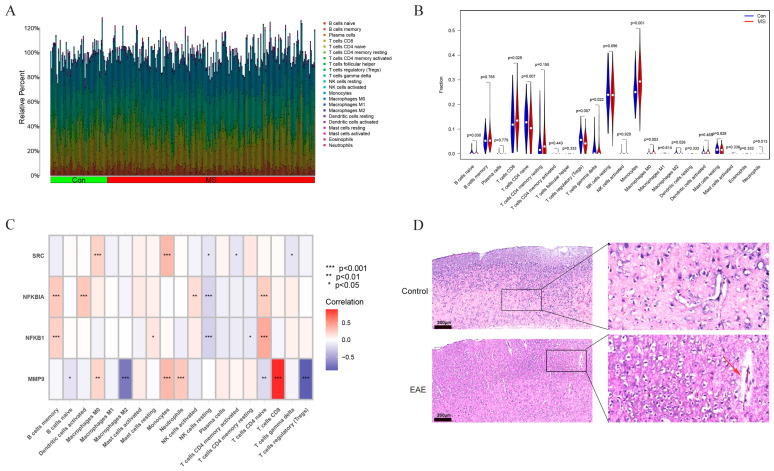
Infiltrative immune cell spectrum and related analysis of MS samples: (**A**) comparison of the percentages of 21 different types of immune cells infiltrating the MS samples and normal samples; (**B**) differences in the proportions of 21 different types of immune cells between the MS and normal samples; (**C**) correlations between the 4 feature genes and immune cells; (**D**) leukocyte infiltration in the cerebral cortex of EAE mice. Scale bars = 200 μm.

In MS, the interplay between immune responses and oxidative stress drives disease progression. Using the CIBERSORT method, we further validated the association between the expression of characteristic genes and immune components. In patients with MS, we constructed a profile of 21 different types of immune cells and analyzed the proportions of these immune cell subtypes (Figure 7A). Our study reveals that several immune cells displayed dysregulated levels between the MS samples and normal samples, such as naive B cells, CD8 T cells, CD4 naive T cells, regulatory T cells (Tregs), gamma delta T cells, monocytes, M0 macrophages, and M2 macrophages (Figure 7B). Further analysis showed that *MMP9* levels were positively correlated with M0 macrophages, monocytes, neutrophils, and CD8 T cells but negatively correlated with M2 macrophages and regulatory T cells (Tregs) (Figure 7C). *NFKB1* levels were positively correlated with memory B cells, resting mast cells, and CD4 naive T cells but negatively correlated with resting NK cells (Figure 7C). *NFKBIA* levels were positively correlated with memory B cells, activated dendritic cells, activated NK cells, and CD4 naive T cells but negatively correlated with resting NK cells (Figure 7C). *SRC* levels were positively correlated with M0 macrophages and monocytes but negatively correlated with resting NK cells (Figure 7B). Additionally, *NFKBIA* levels were positively correlated with activated dendritic cells, activated NK cells, and CD4 naive T cells but negatively correlated with resting NK cells, CD4 memory activated T cells, and gamma delta T cells (Figure 7C).

**Figure 8:**
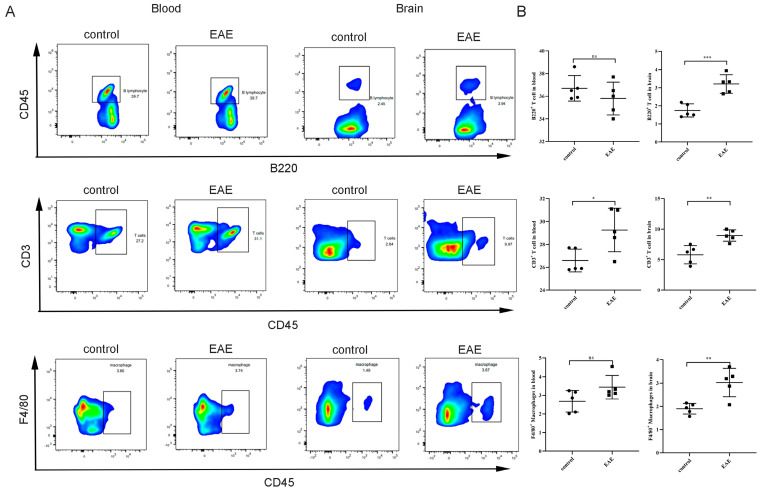
Proportions of immune cells in peripheral blood and brain tissue of the EAE mice: (**A**) flow cytometry analysis of the proportions of B220^+^ B cells, CD3^+^ T cells, and F4/80^+^ macrophages in the peripheral blood and brain of the EAE mice; (**B**) quantitative chart of immune cell proportions in the EAE and normal mice. Compared with the control group: ns *p* > 0.05, * *p* < 0.05, ** *p* < 0.01, and *** *p* < 0.001.

In order to delve into the infiltration of immune cells in the EAE mouse model, we conducted H&E staining on mouse brain tissues. The results reveal a significant presence of leukocyte infiltration in the cortex of the EAE mice, while no notable abnormalities were observed in normal mice (Figure 7D). Additionally, to further determine whether there was dysregulation of immune cell subtypes in the EAE mouse model, we performed a flow cytometry analysis on cells from both blood and brain tissues. The results show a significant increase in the proportions of B220+ B cells, CD3+ T cells, and F4/80+ macrophages in the brain tissues of the EAE mice (Figure 8A,B). Furthermore, in the peripheral blood of the EAE mice, except for CD3+ T cells, no significant differences were observed in the proportions of B220+ B cells and F4/80+ macrophages compared to the normal mice (Figure 8A,B).

### 2.7. Feature Genes and Small Molecular Compound Docking

**Figure 9:**
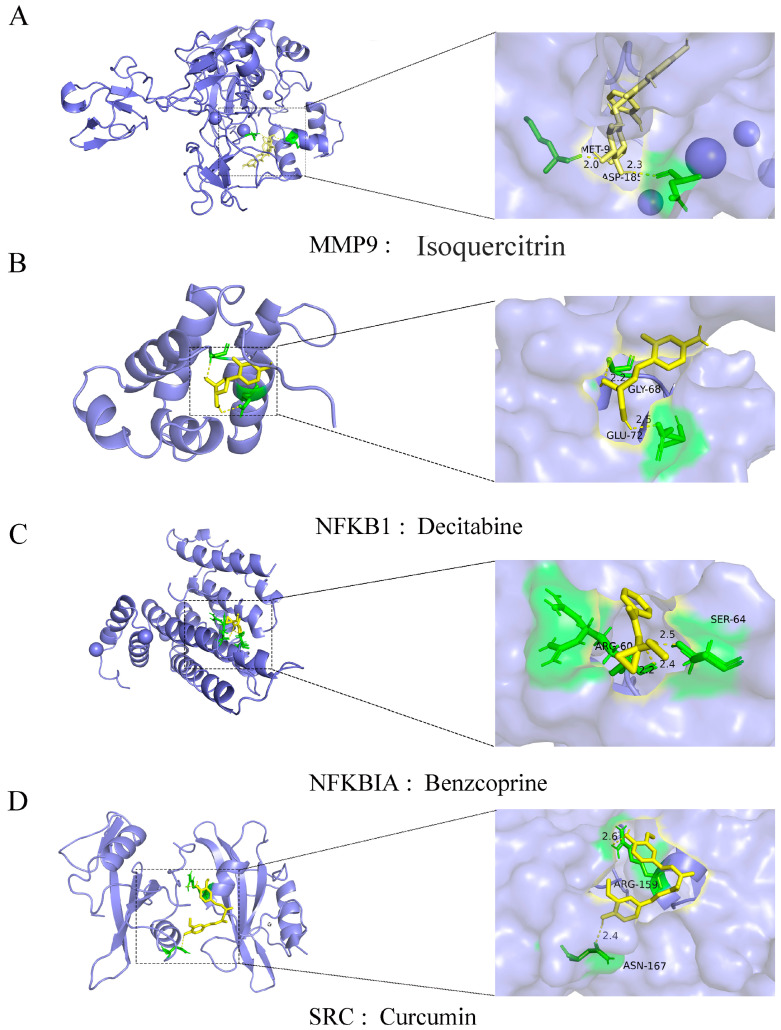
Molecular docking results of the proteins encoded by the feature genes with small molecule compounds: (**A**) docking result of *MMP9* with isoquercitrin; (**B**) docking result of *NFKB1* with decitabine; (**C**) docking result of *NFKBIA* with benztropine; (**D**) docking result of *SRC* with curcumin.

Subsequently, we employed the CTD database, pharmacological research, and automated molecular docking techniques to explore drugs targeting characteristic genes. Initially, we observed that isoquercitrin can tightly bind to *MMP9* and diminish its expression (Figure 9A). The molecular docking analysis indicates that isoquercitrin may interact with MMP9, forming a highly favorable docking binding energy of −8.0 (kcal/mol). Decitabine, a DNA methyltransferase inhibitor, exhibited the ability to effectively reduce *NFKB1* expression in our investigation. By inhibiting DNA methyltransferase activity, Decitabine potentially induces DNA demethylation of the *NFKB1* gene, thereby augmenting *NFKB1* gene transcription and NF-κB1 protein synthesis (Figure 9B). However, further studies are warranted to ascertain the efficacy of decitabine in MS treatment. Benztropine, an anticholinergic drug, mitigates symptoms of degenerative diseases like muscle stiffness and tremors by blocking cholinergic neurotransmission in the central nervous system. Our findings suggest that benztropine can bind to *NFKBIA*, impeding it with a high docking energy of −6.1 (kcal/mol) (Figure 9C). Additionally, curcumin, a natural compound derived from turmeric root, possesses diverse biological activities. Extensively researched for its therapeutic potential, Curcumin exhibits binding affinity to *SRC*, with a docking binding energy reaching −5.7 (kcal/mol) (Figure 9D).

## 3. Discussion

The primary aim of this study was to elucidate the pathophysiological mechanisms underlying MS and identify potential molecular markers and therapeutic targets. By integrating the microarray expression profiles and conducting comprehensive bioinformatics analyses, we discovered significant gene expression differences between patients with MS and control groups. We identified key OSRGs and DEGs, highlighting the crucial role of oxidative stress in MS pathophysiology. Moreover, we established a diagnostic prediction model using key genes, which demonstrated high diagnostic accuracy and clinical feasibility. These findings provide valuable insights into the molecular mechanisms of MS and offer potential avenues for early diagnosis and targeted therapy.

MS is a prevalent chronic disorder of the central nervous system, leading to the demyelination of neurons, axonal damage, and pathological changes in glial cells [16,17]. These pathological alterations can result in irreversible neurological damage, posing a serious threat to human life and health [17]. The limited understanding of the pathophysiological mechanisms and treatment strategies for MS underscores the urgent need to discover new molecular markers and potential therapeutic targets. Furthermore, MS manifests as multifocal, temporally scattered lesions in the central nervous system, encompassing demyelination, axonal severance, and neuronal death [18,19]. The frequent misdiagnosis and treatment challenges of MS often result in poor patient prognosis, which may significantly contribute to the adverse outcomes [20]. As MS is the outcome of complex interactions among multiple genes, its molecular mechanisms remain poorly understood. Thus, identifying effective biomarkers for early diagnosis and targeted therapy is critically needed.

The pathological characteristics of MS include demyelination, axonal damage, inflammatory infiltration, and gliosis [21,22]. While the precise pathogenesis of MS remains unclear, a growing body of research indicates that oxidative stress and inflammatory responses are key drivers of MS progression [23,24]. Chronic local inflammatory stress not only directly causes tissue damage and cell death but also impairs antioxidant defenses, perpetuating a vicious cycle [25,26]. In essence, the progression of MS is marked by the interaction between oxidative stress and inflammation. In our study, we analyzed microarray expression profiles and discovered significant gene expression differences between patients with MS and control groups. This suggests that the pathogenesis of MS may be tightly linked to dysregulation of gene expression. Furthermore, we identified overlapping genes between OSRGs and DEGs, encompassing a series of genes implicated in MS development. These findings imply that oxidative stress plays a crucial role in MS pathophysiology, offering valuable insights for uncovering the mechanisms underlying MS. The functional enrichment analysis revealed that the DE-OSRGs are primarily involved in several biological processes, including intrinsic apoptotic signaling pathways, regulation of inflammatory responses, and responses to oxidative stress—all of which are intimately connected to MS pathogenesis. Previous research has demonstrated that neutrophil extracellular trap (NET) formation is crucial in inflammatory and autoimmune diseases and is closely linked to oxidative stress [27]. Oxidative stress enhances NET formation which, in turn, triggers inflammatory responses and worsens disease progression [28]. Consequently, the enrichment of DE-OSRGs in the NET formation pathway may further intensify inflammatory responses and disease progression. Moreover, functional enrichment analysis has reaffirmed previous findings, underscoring the significance of oxidative-stress-mediated processes, such as immune responses, inflammation, and apoptosis, in the pathogenesis of MS [29].

MS, as an autoimmune disease, benefits significantly from early diagnosis, which allows for timely intervention to alleviate disease progression and mitigate neurological damage, thus improving patients’ quality of life [30]. While some clinical diagnostic biomarkers have been identified, their diagnostic accuracy and clinical feasibility still pose limitations [31]. Therefore, the discovery of new diagnostic biomarkers and the establishment of more accurate diagnostic prediction models are crucial for enhancing the efficiency and accuracy of MS diagnosis. Here, we employed methods such as PPI network construction and LASSO regression to identify four key genes. Subsequently, we developed an MS diagnostic prediction model by integrating these key genes and risk scores. The effectiveness and reliability of this model were validated through calibration curves, ROC curves, and decision curve analysis (DCA), indicating its high diagnostic accuracy and clinical feasibility. *MMP9* is a crucial protease that plays key roles in biological processes such as inflammation [32], cell migration [33], and matrix degradation [34]. Numerous studies have shown that *MMP9* expression levels significantly increase in MS lesion areas, closely correlating with disease severity and progression [35,36]. Therefore, *MMP9* may serve as an important biomarker for assessing the severity of MS and predicting disease progression. *NFKBIA* is a protein that negatively regulates the NF-κB signaling pathway. Inhibition of *NFKBIA* expression can mitigate ROS-induced oxidative stress [37], indicating a potential role for NFKBIA in the activation of the immune system and the mediation of inflammatory responses. *SRC* has been found to be closely associated with neuronal damage and inflammatory responses, participating in the occurrence and development of degenerative diseases [38,39]. The protein encoded by the *NFKB1* gene is an essential component of the NF-κB signaling pathway. NF-κB is a transcription factor crucial for regulating biological processes such as immune and inflammatory responses [40], cell proliferation, and apoptosis [41]. Previous studies have found a significant increase in *NFKB1* expression levels in the nervous system, participating in the immunoinflammatory processes of the disease [42]. Therefore, these genes may serve as important biomarkers for assessing the severity of MS and predicting disease progression.

The EAE model serves as a crucial tool in studying MS, effectively simulating the pathological and clinical features of MS [43]. Our research demonstrates severe clinical symptoms and significant weight loss in EAE mice, accompanied by activated oxidative stress reactions in serum and brain tissues. This suggests an imbalance in oxidative stress responses in the MOG_35–55_-induced EAE mouse model. This finding is consistent with previous research indicating the significant role of oxidative stress in the pathological mechanisms of MS [44]. Through qPCR analysis of the transcription levels of four genes, *MMP9*, *NFKBIA*, *NFKB1*, and *SRC*, in brain tissues, these genes were found to be significantly upregulated in EAE mice, further supporting their importance in the pathological mechanisms of MS. To explore the relationship between these genes and immune cell infiltration, we employed the CIBERSORT method to analyze immune cell infiltration in patients with MS. The results revealed significant differences in multiple immune cell subgroups between MS and normal samples. Specifically, the expressions of *MMP9*, *NFKBIA*, *NFKB1*, and *SRC* were significantly correlated with the proportions of various immune cell subtypes. For instance, *MMP9* levels were positively correlated with M0 macrophages, monocytes, neutrophils, and CD8^+^ T cells while negatively correlated with M2 macrophages and regulatory T cells (Tregs). Immune cell infiltration is another important aspect of the pathological process of MS. Common infiltrating immune cells in the CNS of patients with MS include T cells, B cells, macrophages, and neutrophils [45], which not only participate in inflammatory responses but also exacerbate neuronal damage by releasing cytokines and oxidative products [46]. These results suggest that the *MMP9*, *NFKBIA*, *NFKB1*, and *SRC* genes may influence the pathological process of MS by regulating different immune cell subgroups. Additionally, we observed significant leukocyte infiltration in the cortex of EAE mice, with a significant increase in the proportions of B220^+^ B cells, CD3^+^ T cells, and F4/80^+^ macrophages in the tissue, while no significant differences were observed in peripheral blood. This indicates that immune cell infiltration in the EAE model mainly occurs in the central nervous system, with minimal changes in peripheral blood immune cells.

In the contemporary landscape of medical research, the repositioning of drug functions emerges as a pioneering strategy in disease therapeutics. With the continuous unraveling of MS mechanisms and the ongoing refinement of treatment protocols, a myriad of pharmaceutical agents are being repurposed to tackle and mitigate disease progression [47]. Therefore, anchored on this strategic premise, we embarked on targeted drug screening for diagnostic genes to propose a novel therapeutic paradigm aimed at curbing oxidative-stress-associated MS advancement. In this study, we observed that isoquercitrin demonstrated a remarkable ability to bind tightly to *MMP9*, thereby precipitating a reduction in its expression levels. Moreover, given *MMP9*’s pivotal involvement in a spectrum of maladies including inflammation and cancer [48,49], isoquercitrin emerges as a promising therapeutic agent for these conditions. Decitabine, functioning as a DNA methyltransferase inhibitor [50], exerts a robust suppressive effect on *NFKB1* expression, hypothesized to instigate DNA demethylation of the *NFKB1* gene via the inhibition of DNA methyltransferase activity, thereby fostering enhanced *NFKB1* gene transcription and NF-κB1 protein synthesis [51]. This mechanistic insight positions decitabine as a potential candidate for therapeutic interventions in autoimmune afflictions such as MS. The revelation that benzethonium chloride interfaces with *NFKBIA* unveils a novel therapeutic modality, shedding light on its therapeutic efficacy. Additionally, curcumin, a natural compound extracted from turmeric root, boasts a multifaceted pharmacological profile including antioxidant, anti-inflammatory, and anticancer attributes [52]. The burgeoning body of research underscores curcumin’s promise in the prophylaxis and treatment of diverse ailments [53,54]. In the context of our investigation, curcumin’s interaction with SRC protein unveils a potential mechanism underpinning its anti-inflammatory and anticancer effects by modulating SRC-associated signaling pathways.

In summary, this study revealed significant differences in gene expression, particularly oDE-OSRGs, between patients with MS and normal controls through the integration of various analytical methods. Functional enrichment analysis identified the important roles of these genes in regulating apoptosis signaling, inflammatory responses, and oxidative stress. The constructed diagnostic prediction model and molecular docking analysis demonstrated the potential value of genes such as *MMP9*, *NFKBIA*, *NFKB1*, and *SRC* in the diagnosis and treatment of MS. Additionally, small molecule drugs such as quercetin, decitabine, benzethonium chloride, and curcumin showed high affinity binding to these key genes, providing new therapeutic avenues. These findings provide important insights for both the pathological mechanism research and clinical application of MS.

## 4. Materials and Methods

### 4.1. Data Sources

**Figure 10:**
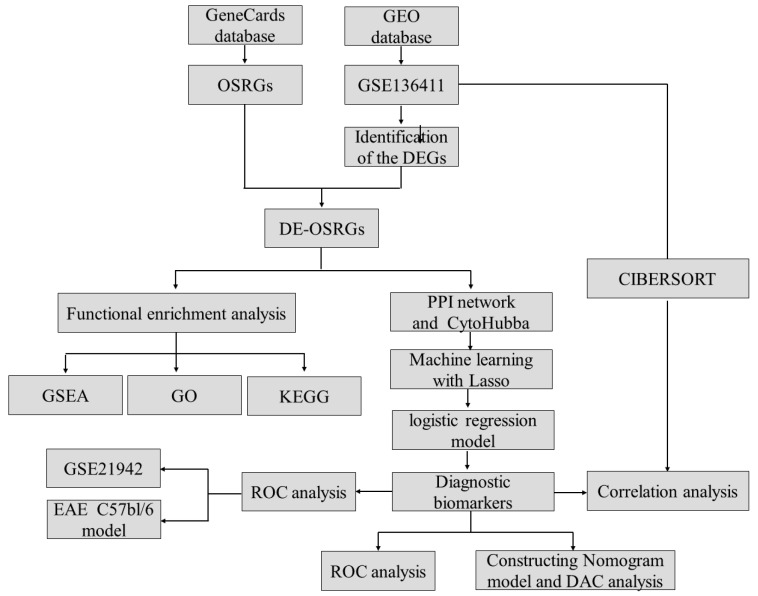
The research methods and design flowchart. GEO: Gene Expression Omnibus; OSRGs: oxidative-stress-related genes; DEGs: differentially expressed genes; DE-OSRGs: differentially expressed oxidative-stress-related genes; PPI: protein–protein interaction; GSEA: gene set enrichment analysis; GO: Gene Ontology; KEGG: Kyoto Encyclopedia of Genes and Genomes; EAE: experimental autoimmune encephalomyelitis.

The overall design of this study is illustrated in Figure 10. To retrieve the mRNA expression data for multiple sclerosis (MS), the search phrases “Multiple sclerosis” and “expression profiling by array” were used. The gene expression microarray datasets GSE136411 and GSE21942 were selected and downloaded from the Gene Expression Omnibus (GEO) database. Additionally, 1399 oxidative-stress-related genes with a relevance score of ≥7 were downloaded from the GeneCards database “https://www.genecards.org” (accessed on 5 March 2024) (Supplementary Table S1).

### 4.2. Identification of Differentially Expressed Genes (DEGs)

The “Limma” (version 4.4.1) software package was employed to conduct differential expression analysis of MS group and control group samples in the GSE136411 dataset. Considering the dataset comprised microarray data with generally small logFC values, the threshold for the selection of DEGs was set at an adjusted *p*-value (p.adj) < 0.05. Given the large number of genes in the original dataset, Benjamini–Hochberg (BH) correction was utilized for multiple testing to control the false discovery rate of the overall significant genes. The adjusted *p*-value achieved a good balance between identifying statistically significant genes and limiting false positives. Subsequently, significant differentially expressed genes associated with oxidative stress (OS) response in MS were identified through overlap analysis between DEGs and 1399 oxidative-stress-related genes.

### 4.3. Pathway Enrichment Analysis

To elucidate potential gene functional annotations and pathway enrichments associated with common differentially expressed oxidative-stress-related genes (DE-OSRGs), Gene Ontology (GO) and Kyoto Encyclopedia of Genes and Genomes (KEGG) analyses were conducted using the “clusterProfiler” software package (version 3.10.1) to showcase potential gene functional annotations. The “enrichplot” package was utilized to visualize the enrichment results. Additionally, gene set enrichment analysis (GSEA analysis) was performed using the “clusterProfiler” package in R (version 4.4.1) based on a standardized gene expression matrix, with pathway gene sets downloaded from the Molecular Signatures Database (MSigDB). Standardized enrichment scores (NES) and p.adjust were respectively used to quantify enrichment magnitude and statistical significance.

### 4.4. Optimal Diagnostic Biomarker Selection and Model Building

The protein–protein interaction (PPI) network of candidate hub genes was constructed using the STRING website, and core network genes were selected with the “cytohubba” plugin in Cytoscape (version 3.10.1). To optimize the data, the LASSO method combined with the “glmnet” package was employed. Based on core network genes, the LASSO algorithm identified gene biomarkers associated with MS. Concurrently, a logistic regression model was built using the predict function from the “glm” package in R, calculating risk scores with biomarkers showing *p* < 0.05 as marker genes. A nomogram was then constructed with risk scores and marker genes as variables. The model’s diagnostic capability was assessed using ROC curves and DAC. Candidate genes consistently reliable across all datasets were selected as the final hub genes. The ROC analysis on the GSE21942 dataset’s samples further validated these genes.

### 4.5. Immune Infiltration Analysis

CIBERSORT (version 4.4.1) is a computational method that infers the number and types of infiltrating immune cells in complex tissues from gene expression data. By referencing specific gene expression matrices, CIBERSORT can accurately estimate the relative proportions of various immune cells in mixed cell populations. We used CIBERSORT to determine the immune cell proportions in the MS and normal groups and evaluated the correlation between hub genes and these immune cell types.

### 4.6. EAE Model and Scoring

All procedures were approved by the Ethics Committee of Kunming Medical University. Female C57BL/6 mice, 8–10 weeks, 20 ± 2 g, were housed with a 12 h light–dark cycle. Disease induction followed previously reported methods [55]. In essence, the mice were inoculated with myelin oligodendrocyte glycoprotein (MOG) (Sigma, St. Louis, MO, USA) to establish a model of primary progressive multiple sclerosis. MOG_35–55_ was emulsified in complete Freund’s adjuvant supplemented with inactivated Mycobacterium tuberculosis H37Ra. As depicted in Figure 6A, on day zero, mice were subcutaneously injected with 200 μg of MOG emulsion, followed by intraperitoneal injection of 200 ng pertussis toxin (PTX, Sigma) diluted in sterile PBS within 3 h; 48 h later, a second 200 ng PTX booster injection was administered. Clinical symptoms of EAE in mice were scored daily according to the following criteria [56]: 0, no clinical disease; 1, tail weakness; 2, ataxia; 3, hind limb paralysis; 4, forelimb paralysis; 5, death.

### 4.7. Chemical Index Detection

Blood samples were obtained from the posterior ethmoid sinus, and brain tissue samples were ground into suspension. The levels of MDA and SOD were assessed using commercial assay kits to evaluate oxidative stress levels. Following the manufacturer’s instructions, samples and standards were incubated with different concentrations of reagents. Finally, absorbance values were measured using a full spectrum microplate reader, and the activities of MDA and SOD in the samples were calculated using standard curves.

### 4.8. RNA Extraction and qRT-PCR

Total mRNA was extracted using Trizol reagent (Invitrogen, CA, USA). For cDNA synthesis, 1 μg of total mRNA was reverse transcribed using the PrimeScript™ RT reagent Kit (Takara, Shiga, Japan). Real-time quantitative PCR analysis was conducted using the TB Green^®^ Premix Ex Taq™ II FAST qPCR assay kit (Takara, Shiga, Japan). The primer sequences, including TNFα, IL-1b, IL-17a, and IFN, are provided in Supplementary Table S2. Each target underwent duplicate analysis. The average mRNA levels were normalized to β-actin mRNA levels as an internal control.

### 4.9. H&E Staining

H&E staining was used to assess the level of inflammatory cell infiltration. Five mice from each group were euthanized, and their brains were immediately extracted and fixed in 4% paraformaldehyde for 24 h. The brain tissues were then dehydrated and embedded in paraffin. Thin sections were prepared using a microtome, and H&E staining was performed according to standard procedures to examine leukocyte infiltration in the cortical vessels.

### 4.10. Flow Cytometry Analysis

The method for isolating single-nucleus cells from brain tissue has been extensively described in previous literature [57]. In the experiment, mice were euthanized under deep isoflurane anesthesia, followed by perfusion through the heart with 4 °C cold 1× PBS to remove leukocytes from the vasculature. The dissected brain tissue was homogenized in Hank’s Balanced Salt Solution (HBSS) (Gibco, NY, USA) using a tissue homogenizer, and then filtered through a 70 μm cell strainer. After centrifugation, the homogenate pellet was resuspended in 40% isotonic Percoll solution (GE-Healthcare, IL, USA) and slowly overlaid with 70% isotonic Percoll solution. Percoll density gradient was formed by centrifugation at 500 *g* for 30 min. The mononuclear layer was collected from the interface of the Percoll solution. After washing with 1x PBS, interfering leukocytes were excluded by PerCP-anti-CD45 (Biolegend, CA, USA) labeling. To detect the proportions of B cells, T cells, and mature macrophages, cells were stained using Alexa Fluor^®^ 488-anti-B220 (Biolegend, CA, USA), FITC-anti-CD3 (Biolegend, CA, USA), APC-anti-F4/80 (Biolegend, CA, USA), and APC-anti-CD80 (Biolegend, CA, USA). The representative gating strategy for the experiment is shown in Supplementary Figure S1.

### 4.11. Drugs Screened and Docking

Based on the functional analysis of characteristic genes, protein-coding genes were selected for targeted drugs. The criteria for drug selection focused on the expression of characteristic genes in patients with MS. First, a catalog of small molecule compounds interacting with the selected genes was obtained from the CTD database “http://ctdbase.org/” (accessed on 3 April 2024) and their structures from the PDB database “https://rcsb.org/” (accessed on 4 April 2024). Next, the structures of the selected genes’ biomacromolecules were downloaded from the Uniprot database “https://uniprot.org/” (accessed on 4 April 2024). Finally, automatic docking of biomacromolecules and small molecule compounds was performed using vina based on the standard docking procedure. The interactions between small molecule compounds and biomacromolecules were determined by the lowest binding energy. The results were visualized using PyMol (version 3.0).

### 4.12. Statistical Analysis

All data processing and analyses were performed using R version 4.1.3. Spearman rank correlation analysis was used to assess the correlation between two continuous variables. The Student’s *t*-test and chi-square test were employed to investigate and compare the results among groups. The results were considered statistically significant when *p* < 0.05.

## Data Availability

Data is contained within the article and Supplementary Materials.

## Supplementary Materials

The following supporting information can be downloaded at: https://www.mdpi.com/article/10.3390/ijms25147551/s1.
