# Supplementary material for: Identifying Diagnostic Markers and Constructing Predictive Models for Oxidative Stress in Multiple Sclerosis
Source: Int J Mol Sci. 2024 Jul 10;25(14):7551. doi: 10.3390/ijms25147551 (PMC11276709; doi:10.3390/ijms25147551)
Supplement: Supplementary file 1 [file ijms-25-07551-s001.zip › Supplemental caption.pdf]

Supplemental Table S1: 1399 oxidative stress-related genes

Supplemental Table S2: The primer sequences for the four hub genes.

Supplemental Figure S1: Representative gating strategy for flow cytometry analysis.
