# Supplementary material for: Identifying Diagnostic Markers and Constructing Predictive Models for Oxidative Stress in Multiple Sclerosis
Source: Int J Mol Sci. 2024 Jul 10;25(14):7551. doi: 10.3390/ijms25147551 (PMC11276709; doi:10.3390/ijms25147551)
Supplement: Supplementary file 1 [file ijms-25-07551-s001.zip › Supplemental table S2.pdf]

The primer sequences for the four hub genes

Genes	Primers	Sequences
MMP9	Sense	Antisense
	TCACCATGAGTCCCTGGCA	AGCGGTACAAGTATGCCTCTGC
NFKB1	Sense	Antisense
	CCACAAGGGGACATGAAGCA	TCCCGGAGTTCATCTCATAGT
NFKBIA	Sense	Antisense
	AGAACAACCTGCAGCAGACTC	TTCCTCGAAAGTCTCGGAGC
SRC	Sense	Antisense
	TCTGAACCAAGGCAGCATCT	TGGCCTAAAGACCCTGTTGC
