# Supplementary figures and images for: Identifying Diagnostic Markers and Constructing Predictive Models for Oxidative Stress in Multiple Sclerosis
Source: Int J Mol Sci. 2024 Jul 10;25(14):7551. doi: 10.3390/ijms25147551 (PMC11276709; doi:10.3390/ijms25147551)

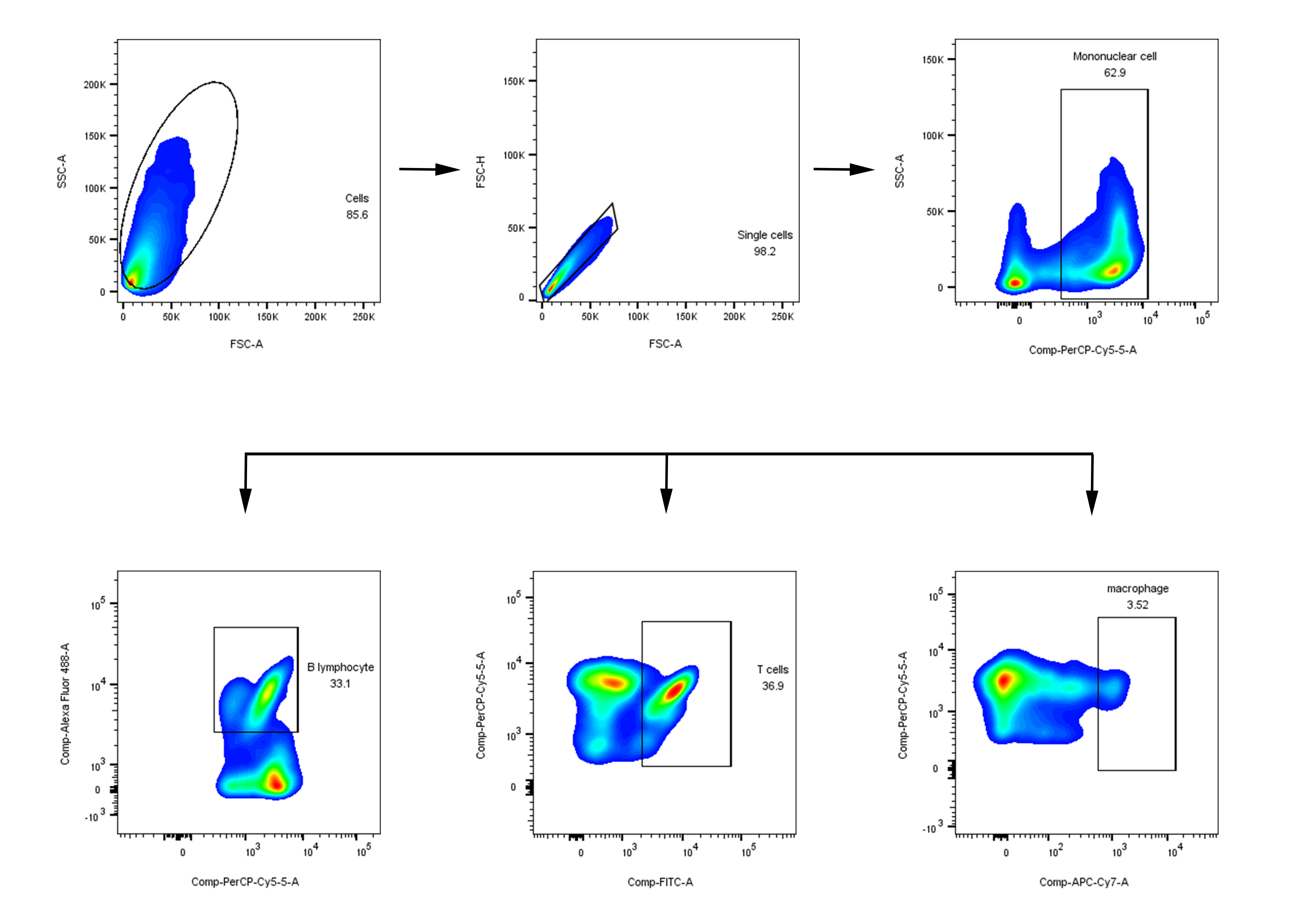

Supplement: Supplementary file 1 [file ijms-25-07551-s001.zip › Supplemental Figure S1.tif]
